# Cumulative dose of epinephrine and mode of death after non-shockable out-of-hospital cardiac arrest: a registry-based study

**DOI:** 10.1186/s13054-023-04776-0

**Published:** 2023-12-20

**Authors:** François Javaudin, Wulfran Bougouin, Lucie Fanet, Jean-Luc Diehl, Daniel Jost, Frankie Beganton, Jean-Philippe Empana, Xavier Jouven, Frédéric Adnet, Lionel Lamhaut, Jean-Baptiste Lascarrou, Alain Cariou, Florence Dumas, the Sudden Death Expertise Center investigators, F. Adnet, J. M. Agostinucci, N. Aissaoui-Balanant, V. Algalarrondo, F. Alla, C. Alonso, W. Amara, D. Annane, C. Antoine, P. Aubry, E. Azoulay, F. Beganton, C. Billon, W. Bougouin, J. Boutet, C. Bruel, P. Bruneval, A. Cariou, P. Carli, E. Casalino, C. Cerf, A. Chaib, B. Cholley, Y. Cohen, A. Combes, J. M. Coulaud, M. Crahes, D. Da Silva, V. Das, A. Demoule, I. Denjoy, N. Deye, J. L. Diehl, S. Dinanian, L. Domanski, D. Dreyfuss, D. Duboc, J. L. Dubois-Rande, F. Dumas, J. Duranteau, J. P. Empana, F. Extramiana, J. Y. Fagon, M. Fartoukh, F. Fieux, M. Gabbas, E. Gandjbakhch, G. Geri, B. Guidet, F. Halimi, P. Henry, F. Hidden Lucet, P. Jabre, L. Joseph, D. Jost, X. Jouven, N. Karam, H. Kassim, J. Lacotte, K. Lahlou-Laforet, L. Lamhaut, A. Lanceleur, O. Langeron, T. Lavergne, E. Lecarpentier, A. Leenhardt, N. Lellouche, V. Lemiale, F. Lemoine, F. Linval, T. Loeb, B. Ludes, C. E. Luyt, A. Maltret, N. Mansencal, N. Mansouri, E. Marijon, J. Marty, E. Maury, V. Maxime, B. Megarbane, A. Mekontso-Dessap, H. Mentec, J. P. Mira, X. Monnet, K. Narayanan, N. Ngoyi, M. C. Perier, O. Piot, R. Pirracchio, P. Plaisance, B. Plaud, I. Plu, J. H. Raphalen, M. Raux, F. Revaux, J. D. Ricard, C. Richard, B. Riou, F. Roussin, F. Santoli, F. Schortgen, A. Sharifzadehgan, T. Sharshar, G. Sideris, T. Similowski, C. Spaulding, J. L. Teboul, J. F. Timsit, J. P. Tourtier, P. Tuppin, C. Ursat, O. Varenne, A. Vieillard-Baron, S. Voicu, K. Wahbi, V. Waldmann

**Affiliations:** 1 Paris Sudden Death Expertise Center, 75015 Paris, France; 2 Emergency Department, Nantes University Hospital, 44000 Nantes, France; 3 Université Paris Cité, INSERM U970, Paris Cardiovascular Research Center (PARCC), European Georges Pompidou Hospital, 75015 Paris, France; 4 Medical Intensive Care Unit, Ramsay Générale de Santé, Hôpital Privé Jacques Cartier, 6 Avenue du Noyer Lambert, 91300 Massy, France; 5 AfterROSC Network, Paris, France; 6 Medical Intensive Care Unit, AP-HP, European Georges Pompidou Hospital, 75015 Paris, France; 7 Innovative Therapies in Hemostasis, INSERM 1140, Université Paris Cité, 75006 Paris, France; 8 BSPP (Paris Fire-Brigade Emergency-Medicine Department), 1 Place Jules Renard, 75017 Paris, France; 9 Cardiology Department, AP-HP, European Georges Pompidou Hospital, 75015 Paris, France; 10 SAMU de Paris, Necker University Hospital, Assistance Publique-Hôpitaux de Paris, 75015 Paris, France; 11 Medecine Intensive Reanimation, Nantes University Hospital, 44000 Nantes, France; 12 Medical Intensive Care Unit, AP-HP, Cochin Hospital, 75014 Paris, France; 13 Emergency Department, AP-HP, Cochin-Hotel-Dieu Hospital, 75014 Paris, France; 14 SAMU, 1 Quai Moncousu, 44093 Nantes Cedex1, France

**Keywords:** Epinephrine, Cardiac arrest, Non-shockable OHCA, Cardiocirculatory death, Post-resuscitation shock

## Abstract

**Background:**

Epinephrine increases the chances of return of spontaneous circulation (ROSC) in out-of-hospital cardiac arrest (OHCA), especially when the initial rhythm is non-shockable. However, this drug could also worsen the post-resuscitation syndrome (PRS). We assessed the association between epinephrine use during cardiopulmonary resuscitation (CPR) and subsequent intensive care unit (ICU) mortality in patients with ROSC after non-shockable OHCA.

**Methods:**

We used data prospectively collected in the Sudden Death Expertise Center (SDEC) registry (capturing OHCA data located in the Greater Paris area, France) between May 2011 and December 2021. All adults with ROSC after medical, cardiac and non-cardiac causes, non-shockable OHCA admitted to an ICU were included. The mode of death in the ICU was categorized as cardiocirculatory, neurological, or other.

**Results:**

Of the 2,792 patients analyzed, there were 242 (8.7%) survivors at hospital discharge, 1,004 (35.9%) deaths from cardiocirculatory causes, 1,233 (44.2%) deaths from neurological causes, and 313 (11.2%) deaths from other etiologies. The cardiocirculatory death group received more epinephrine (4.6 ± 3.8 mg versus 1.7 ± 2.8 mg, 3.2 ± 2.6 mg, and 3.5 ± 3.6 mg for survivors, neurological deaths, and other deaths, respectively; *p* < 0.001). The proportion of cardiocirculatory death increased linearly (*R*^2^ = 0.92, *p* < 0.001) with cumulative epinephrine doses during CPR (17.7% in subjects who did not receive epinephrine and 62.5% in those who received > 10 mg). In multivariable analysis, a cumulative dose of epinephrine was strongly associated with cardiocirculatory death (adjusted odds ratio of 3.45, 95% CI [2.01–5.92] for 1 mg of epinephrine; 12.28, 95% CI [7.52–20.06] for 2–5 mg; and 23.71, 95% CI [11.02–50.97] for > 5 mg; reference 0 mg; population reference: alive at hospital discharge), even after adjustment on duration of resuscitation. The other modes of death (neurological and other causes) were also associated with epinephrine use, but to a lesser extent.

**Conclusions:**

In non-shockable OHCA with ROSC, the dose of epinephrine used during CPR is strongly associated with early cardiocirculatory death. Further clinical studies aimed at limiting the dose of epinephrine during CPR seem warranted. Moreover, strategies for the prevention and management of PRS should take this dose of epinephrine into consideration for future trials.

**Supplementary Information:**

The online version contains supplementary material available at 10.1186/s13054-023-04776-0.

## Background

The incidence of out-of-hospital cardiac arrest (OHCA) in Europe and the USA is estimated to be between 48 and 170 per 100,000 inhabitants, with an overall survival of 8–9% [1–3]. Nevertheless, this survival rate is much lower in the case of a non-shockable initial rhythm (approximately 2% versus 25% in the case of a shockable rhythm) [4]. Initial non-shockable rhythms represent the majority (around 80%) of OHCAs and have been increasing for several years [4–6]. Furthermore, among non-shockable patients with return of spontaneous circulation (ROSC) admitted to an intensive care unit (ICU), the mortality rate amounts to 90% [4]. The medical causes most frequently identified among patients admitted to ICU after non-shockable cardiac arrest are cardiac cause and asphyxia for non-cardiac causes [7]. The neurological outcome of patients following asphyxial cardiac arrest is poor [8].

During cardiopulmonary resuscitation (CPR) in a non-shockable rhythm, it is recommended to use 1 mg of epinephrine every 3–5 min to increase the chance of ROSC [9, 10]. In this indication, epinephrine improves survival rates but not neurological outcomes [11]. However, many adverse effects are attributed to the use of epinephrine. First, a high dose is associated with post-resuscitation myocardial dysfunction (PRMD) and, consequently, with post-resuscitation syndrome (PRS) [12]. Second, epinephrine promotes arrhythmias, such as re-arrest with shockable rhythm [13]. Third, epinephrine decreases cerebral microvascular blood flow and would be responsible for poor neurological outcomes by increasing cerebral ischemia [14]. These deleterious effects after ROSC are well supported by the fact that the more subjects receive epinephrine during CPR, the more they develop refractory PRS and the lower their survival rate [15–17]. Thus, it seems that the dose of epinephrine used during CPR favors a cardiocirculatory mode of death after ROSC by promoting PRS and re-arrests. However, data on the mode of death after cardiac arrest remain scarce. Witten et al. [18] reported that the most common cause of death after ROSC following cardiac arrest was the neurological withdrawal of life-sustaining therapy (WLST) (73%). In contrast, refractory PRS and sudden cardiac death accounted for 17% and 4%, respectively. The same proportions were found a decade earlier by Laver et al. [19], who reported 67.7% of deaths due to neurological injury, and 65.2% reported by Lemiale et al. [20]. The rate of brain death following conventional CPR is estimated at 8% [21]. Nevertheless, it remains unclear whether the dose of epinephrine administered during CPR directly influences the mode of death after ROSC. Therefore, these data seem crucial to optimize therapeutics and personalize management in the ICU [22]. A more precise identification of the possible effects of epinephrine on different modes of death could allow better management by identifying patients at risk earlier and adopting a more targeted prevention strategy.

The aim of our study was to investigate the association between the cumulative dose of epinephrine used during CPR and the mode of death in the ICU in non-shockable OHCA patients.

## Methods

### Study design and setting

We used data from the cardiac arrest registry managed by the Sudden Death Expertise Center (SDEC) of the Greater Paris area (France). It is a population-based registry that records all OHCA since 2011 occurring in Paris and its suburbs (Hauts-de-Seine, Seine-Saint-Denis, Val-de-Marne), which covers an area of 6.8 million inhabitants on 762 km^2^ (294 square miles) [4]. The Paris-SDEC registry is approved by the French Advisory Committee on Information Processing in Health Research (CCTIRS, authorization no. 12336) and the French National Data Protection Commission (CNIL, authorization no. 912309). No consent was required, and survivors were informed of their inclusion in the register with the possibility of requesting removal. In France, the Emergency Medical Service (EMS) is a two-tiered physician-manned system, with a basic life support (BLS) tier served by firefighters and an advanced life support (ALS) tier provided in the field by a mobile intensive care unit (MICU). The MICU includes at least one ambulance driver, one nurse, and one trained emergency physician. A detailed description of the French EMS has been previously published [23].

### Study population

From May 2011 to December 2021, all non-traumatic OHCAs with initial non-shockable rhythms that achieved prehospital ROSC and admitted to an ICU were included. During this period, the guidelines for the use of epinephrine during CPR of non-shockable rhythms remained unchanged. Exclusion criteria were age below 18 years, traumatic OHCA, death on the scene or during transport, admission to hospital for uncontrolled organ donation, and missing data (cumulative epinephrine dose during CPR, vital status, and mode of death).

### Data collection

All data, including Utstein characteristics of the OHCA [24], were recorded prospectively. These included gender, age, location, presence of witnesses, bystander CPR, resuscitation time intervals (no-flow duration, low-flow duration), initial cardiac rhythm, epinephrine dose during CPR, medical history, ECMO use, occurrence of PRS, presumed cause of cardiac arrest, angioplasty intervention, targeted temperature management (TTM), vital status (obtained at hospital discharge), and mode of death. The epinephrine doses used during CPR were divided into 4 categories after testing for linearity: none, 1 mg, 2–5 mg, and > 5 mg, as previously described [16].

### Endpoints

The primary endpoint was the mode of death in patients admitted to the ICU after non-shockable OHCA. Three categories of the mode of death were retained: i) cardiocirculatory death, including PRS, refractory cardiac arrest, and recurrence of sudden cardiac arrest; ii) neurological death, including brain death and neurological WLST; and iii) other death, including comorbid WLST, respiratory failure, and all death modes not classified in the first two categories. To ensure the accuracy of classification, two investigators independently reviewed hospital records of all cases and assigned to each patient one of these modes of death. All discrepancies were resolved with additional assessment by a third investigator. Patients meeting the criteria for more than one mode of death category were classified according to the primary reason for death. In a previous SDEC work (from 2011 to 2018), we highlighted that the agreement between the two investigators regarding the classification of the mode of death was considered good (kappa coefficient 0.87) [17]. Secondary endpoints were the survival rate and the time to death according to the mode of death.

### Statistical analysis

We used descriptive statistics to summarize categorical variables as proportions and continuous variables as the mean with a standard deviation. Comparisons between proportions used Pearson’s chi-squared test and ANOVA or *t*-test for continuous variables.

We reported the outcome of patients according to the cumulative dose of epinephrine during CPR in 4 categories: alive at hospital discharge, cardiocirculatory death, neurological death, and other causes of death. A univariate analysis was performed to test the different parameters associated with survival and mode of death. Linear regressions between epinephrine dose and cardiocirculatory death rate, and between epinephrine dose and low-flow duration were performed. The association between vital status, mode of death, and cumulative dose of epinephrine (divided into 4 categories: 0 mg, 1 mg, 2–5 mg, and > 5 mg) was analyzed in a multivariable multinomial regression analysis using those who survived as the reference group. It was adjusted for age, gender, location, witnessed OHCA, bystander CPR, no-flow duration, initial electrical rhythm, low-flow duration, presumed cause (cardiac/non-cardiac), and all variables with p values < 0.15 in univariate analysis. A generalized Hosmer–Lemeshow test for multinomial logistic regression was used to evaluate the model [25]. To account for missing data in the primary multivariable analysis, we performed multiple imputations using a chained equation [26]. To assess whether there were differences in outcomes over the study period, we performed sensitivity analyses: including the year of OHCA as one additional covariate, using epinephrine as a continuous variable, and stratifying on the duration of resuscitation (tertiles of low-flow duration).

All tests were two-sided with a *p* value considered significant if < 0.05. Analyses were performed using STATA/SE 15.1 (Lakeway Drive, TX, USA).

## Results

**Fig. 1:**
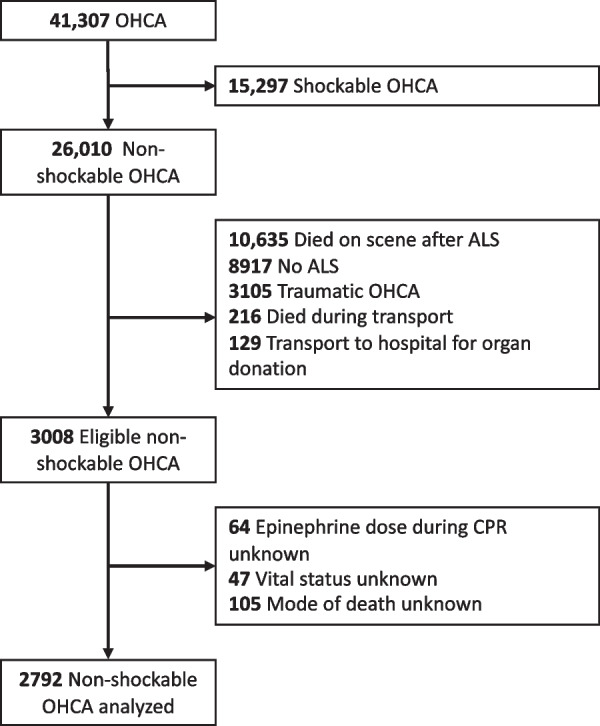
Flowchart of the patient selection. OHCA, out-of-hospital cardiac arrest; ALS, advanced life support; and CPR, cardiopulmonary resuscitation

**Fig. 2:**
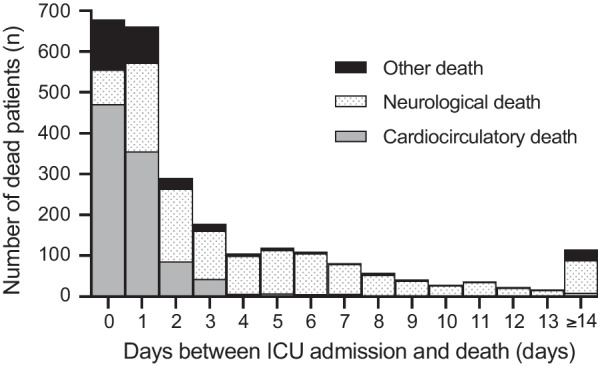
Mode of death according to the delay between ICU admission and death. ICU, intensive care unit

From May 2011 to December 2021, there were 41,307 cases of OHCA in the Paris-SDEC database. After screening for eligibility, 3,008 patients were initially retained in the analysis. Because of missing data in 216 subjects, a total of 2,792 OHCAs could be analyzed (Fig. 1). The mean age was 63.5 ± 15.3 years, and 61.0% were male. There were 242 (8.7%) survivors at hospital discharge. Among non-survivors, there were 1,004 (35.9%) deaths from cardiocirculatory causes (672 hemodynamic shock, 295 recurrence of sudden cardiac arrest, 37 refractory cardiac arrest), 1,233 (44.2%) deaths from neurological causes (488 brain deaths and 745 neurological WLST), and 313 (11.2%) deaths from other etiologies (212 comorbid WLST, 16 respiratory failures, and 85 others). Patients’ characteristics according to their outcome are reported in Table 1, and according to the dose of epinephrine administered during CPR in Table S1 in Additional file 1. As compared with other groups, the cardiocirculatory death group received more epinephrine during CPR (mean 4.6 mg), had longer resuscitation time (mean low-flow duration 36.2 min), presented more frequently with PRS (81.3%), required ECMO more often (13.4%), and the presumed cause of OHCA was more frequently of cardiac origin (62.7%). The time from OHCA to death was significantly shorter in the cardiocirculatory death group (1.2 ± 3.3 days versus 3.7 ± 9.1 and 5.5 ± 8.2 days for the other death group and neurological group, respectively; *p* < 0.001) (Fig. 2). The correlation coefficient (*R*) between epinephrine dose during CPR and low-flow duration was 0.51 (Additional file 1: Figure S1).

**Table 1:** Characteristics of study population

Variables n (%) or mean ± SD	Alive (n = 242)	Cardiocirculatory death (n = 1,004)	Neurological death (n = 1,233)	Other death (n = 313)	*p value * Chi2 or ANOVA	Missing data
Male	161 (66.5%)	628 (62.6%)	734 (59.5%)	179 (57.2%)	0.067	0 (0.0%)
Age (years)	61.2 ± 16.0	64.1 ± 14.9	61.6 ± 14.9	70.5 ± 15.7	< 0.001	0 (0.0%)
Home location	152 (62.8%)	731 (72.9%)	876 (71.1%)	250 (80.1%)	< 0.001	2 (0.1%)
Witnessed	212 (87.6%)	828 (82.8%)	998 (81.0%)	275 (87.9%)	0.006	5 (0.2%)
Bystander CPR	167 (69.6%)	597 (60.4%)	654 (53.2%)	193 (62.7%)	< 0.001	26 (0.9%)
No-flow duration (min)	3.4 ± 5.5	6.1 ± 8.6	7.3 ± 8.2	5.9 ± 7.9	< 0.001	409 (14.6%)
First recorded rhythm						
Asystole PEA	195 (80.6%) 47 (19.4%)	836 (83.3%) 168 (16.7%)	1058 (85.8%) 175 (14.2%)	272 (86.9%) 41 (13.1%)	0.072	0 (0.0%)
Epinephrine during CPR						
0 mg 1 mg 2–5 mg > 5 mg	90 (37.2%) 57 (23.6%) 83 (34.3%) 12 (5.0%)	43 (4.3%) 100 (10.0%) 575 (57.3%) 286 (28.5%)	72 (5.8%) 236 (19.1%) 750 (60.8%) 175 (14.2%)	38 (12.1%) 52 (16.6%) 178 (56.9%) 45 (14.4%)	< 0.001	0 (0.0%)
Low-flow duration (min)	17.2 ± 15.6	36.2 ± 27.6	27.7 ± 17.4	26.7 ± 16.8	< 0.001	315 (11.3%)
Heart disease	70 (28.9%)	274 (27.3%)	349 (28.3%)	114 (36.4%)	0.018	0 (0.0%)
Ischemic cardiopathy	32 (13.2%)	157 (15.7%)	172 (14.0%)	55 (17.6%)	0.31	0 (0.0%)
Cancer	27 (11.2%)	97 (9.7%)	135 (11.0%)	59 (18.9%)	0.001	0 (0.0%)
Renal disease	19 (7.9%)	76 (7.6%)	109 (8.8%)	37 (11.8%)	0.126	0 (0.0%)
High blood pressure	106 (43.8%)	399 (39.7%)	527 (42.7%)	147 (47.0%)	0.122	0 (0.0%)
Diabetes mellitus	49 (20.3%)	244 (24.3%)	272 (22.1%)	83 (26.5%)	0.195	0 (0.0%)
Dyslipidemia	48 (19.8%)	164 (16.3%)	208 (16.9%)	53 (16.9%)	0.635	0 (0.0%)
ECMO Shock ARDS Refractory CA	15 (6.2%) 7 (46.7%) 0 (0.0%) 8 (53.3%)	134 (13.4%) 17 (12.7%) 0 (0.0%) 117 (87.3%)	60 (4.9%) 8 (13.3%) 1 (1.7%) 51 (85.0%)	21 (6.7%) 4 (19.0%) 0 (0.0%) 17 (81.0%)	< 0.001	0 (0.0%)
Coronary angioplasty	36 (14.9%)	107 (10.7%)	91 (7.4%)	22 (7.0%)	< 0.001	31 (1.1%)
TTM	122 (52.4%)	264 (26.8%)	628 (51.6%)	81 (26.7%)	< 0.001	55 (2.0%)
Post-resuscitation shock	142 (62.6%)	761 (81.3%)	754 (64.4%)	185 (62.1%)	< 0.001	160 (5.7%)
Presumed cause of CA						
Cardiac Non-cardiac	99 (40.9%) 143 (59.1%)	629 (62.7%) 375 (37.3%)	511 (41.4%) 722 (58.6%)	172 (55.0%) 141 (45.0%)	< 0.001	0 (0.0%)
Details of non-cardiac causes						
Dyskalemia Pulmonary embolism Intracranial bleeding Asphyxia Drug overdose Other	6 (4.2%) 33 (23.1%) 2 (1.4%) 83 (58.0%) 4 (2.8%) 15 (10.5%)	14 (3.7%) 82 (21.9%) 25 (6.7%) 173 (46.1%) 1 (0.3%) 80 (21.3%)	28 (3.9%) 48 (6.6%) 181 (25.1%) 416 (57.6%) 6 (0.8%) 43 (6.0%)	6 (4.3%) 18 (12.8%) 14 (9.9%) 79 (56.0%) 0 (0.0%) 24 (17.0%)	< 0.001	0 (0.0%)

CPR, cardiopulmonary resuscitation; ALS, advanced life support; PEA, pulseless electrical activity; CA, cardiac arrest; ECMO, extracorporeal membrane oxygenation; TTM, targeted temperature management

**Fig. 3:**
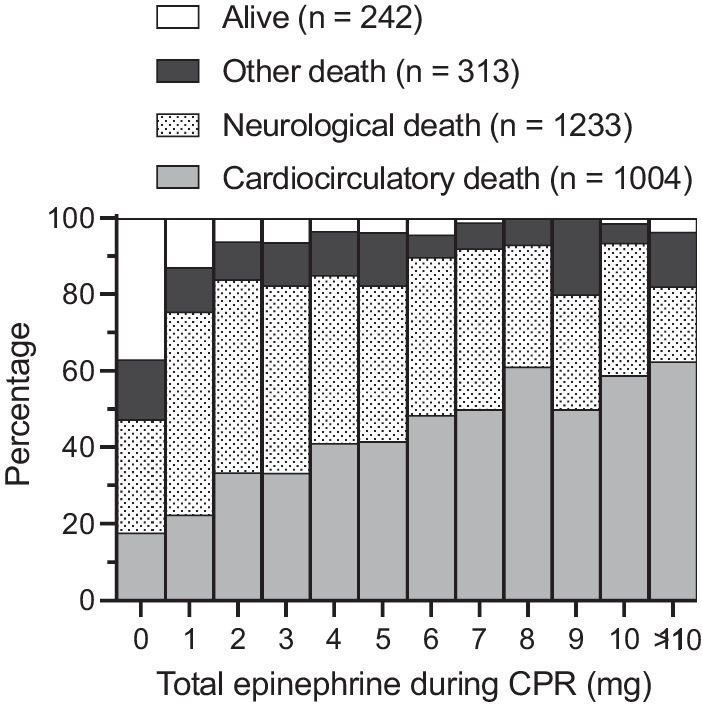
Outcome percentage distribution according to the dose of epinephrine used during CPR. CPR, cardiopulmonary resuscitation

The proportion of cardiocirculatory death increased with cumulative epinephrine doses during CPR (Fig. 3). It represented 17.7% in subjects who did not receive epinephrine and 62.5% in those who received more than 10 mg. There was a linear relationship between the proportion of death from cardiovascular causes and the dose of epinephrine (*R*^2^ = 0.92, *p* < 0.001, Figure S2 in supplemental data). Most survivors (86.8%) were treated with less than or equal to 3 mg of epinephrine during CPR.

The multivariable analysis highlighted a strong association between high-dose epinephrine use and cardiocirculatory death mode (adjusted odds ratio of 3.45, 95% CI [2.01–5.92] for 1 mg of epinephrine to 23.71, 95% CI [11.02–50.97] for > 5 mg). The other modes of death (neurological and other) were also associated with epinephrine use but to a lesser extent (Table 2). Other variables significantly associated with the mode of cardiovascular death were age (aOR 1.04, 95% CI [1.03–1.05] per year), home location (aOR 1.57, 95% CI [1.11–2.24]), no-flow duration (aOR 1.08, 95% CI [1.04–1.12] per minute), low-flow duration (aOR 1.04, 95% CI [1.03–1.06] per minute), high blood pressure history (aOR 0.55, 95% CI [0.38–0.79]), coronary angioplasty intervention (aOR 0.51, 95% CI [0.30–0.86]), TTM (aOR 0.25, 95% CI [0.18–0.35]), PRS (aOR 2.13, 95% CI [1.48–3.05]), and presumed cardiac cause (aOR 2.44, 95% CI [1.69–3.52]). The goodness-of-fit test of this multinomial logistic regression was non-significant (p value = 0.173). The full multivariable model and the analysis without imputation are available in Additional file 1 (Additional file 1: Tables S2 and S3). In the sensitivity analyses, year was not significantly associated with outcome (Additional file 1: Tables S4 and S5); epinephrine as continuous variable was associated with all 3 death modes (aOR 1.46, 95% CI [1.33–1.61] per mg for cardiocirculatory death, aOR 1.33, 95% CI [1.21–1.46] per mg for neurological death, and aOR 1.41, 95% CI [1.27–1.56] per mg for other death; Additional file 1: Table S6). Analyses stratified by tertiles of resuscitation time (0–19 min, 20–30 min, and > 30 min) also showed an association between the dose of epinephrine used during resuscitation and mode of death in all subgroups (Additional file 1: Table S7). The interaction tests between epinephrine and low-flow duration were non-significant in the model (p values > 0.1).

**Table 2:** Multinomial regression testing the association between use of epinephrine and outcomes

	Cardiocirculatory death		Neurological death		Other death	
	aOR 95%CI	p value	aOR 95%CI	p value	aOR 95%CI	p value
Epinephrine during CPR						
0 mg	Reference		Reference		Reference	
1 mg 2–5 mg > 5 mg	3.45 (2.01–5.92) 12.28 (7.52–20.06) 23.71 (11.02–50.97)	< 0.001 < 0.001 < 0.001	4.40 (2.79–6.94) 8.88 (5.80–13.59) 10.26 (4.94–21.31)	< 0.001 < 0.001 < 0.001	2.69 (1.49–4.85) 5.95 (3.49–10.13) 7.45 (3.20–17.33)	0.001 < 0.001 < 0.001

Reference population: alive at hospital discharge

Adjustment variables: gender, age, location, witnessed OHCA, bystander CPR, no-flow duration, initial electrical rhythm, low-flow duration, medical history: heart disease, cancer, renal disease, high blood pressure, ECMO use, coronary angioplasty intervention, TTM, post-resuscitation shock, presumed cardiac cause

aOR, adjusted odds ratio; 95%CI, 95% confidence interval; OHCA, out-of-hospital cardiac arrest; CPR, cardiopulmonary resuscitation; ECMO, extracorporeal membrane oxygenation; TTM, targeted temperature management

## Discussion

This retrospective study is the first to specifically evaluate the mode of death in relation to the dose of epinephrine used during CPR in non-shockable OHCA. We found a strong, independent, and graded association between the risk of early cardiocirculatory death after a non-shockable OHCA and the dose of epinephrine during CPR. Indeed, in patients who received 5 mg or more of epinephrine during CPR, most ICU deaths were due to cardiocirculatory causes.

Several previous studies reported an association between the cumulative dose of epinephrine during CPR and the development of PRMD [27–29]. Paul et al. [30] reported that the majority of subjects with very high Cardiac Arrest Hospital Prognosis (CAHP) scores, which included epinephrine dose and six other variables, were more likely to die of PRS. Binois et al. [17] identified a cluster of patients strongly associated with PRS whose epinephrine dose was notably much higher than others. In this study, we were able to show that this association was not only associated with PRS but also responsible for early mortality from cardiocirculatory causes. In a study including 1,646 subjects admitted to the hospital with ROSC, those who received high doses of epinephrine had a poorer outcome [16]. However, our study did not include patients who died at the scene of OHCA, but only those admitted to ICU with ROSC or ECMO. These results therefore do not contradict the findings of randomized trials reporting that standard-dose epinephrine in non-shockable OHCA increases survival to hospital discharge [31]. However, high-dose epinephrine does not improve survival compared with placebo, whereas it clearly favors ROSC, which means that mortality is increased in the ICU for these patients, as shown in our study. In order to establish a causal link between the use of lower doses of epinephrine and improved outcome, only randomized studies could address this hypothesis. Indeed our observational study only demonstrated an association, not causality. Currently, the CanROC EpiDOSE trial is recruiting and aims to compare a low vs. a standard cumulative dose of epinephrine in shockable OHCAs (32). The LowEPI study will compare reduced epinephrine bolus doses during CPR (0.5 mg) versus standard bolus doses (1 mg) [33]. Indeed, the same team had reported that the coronary perfusion pressures required for ROSC were no different between 0.5 and 1 mg boluses in a porcine model [34].

Data from our study suggest that management after ROSC in the ICU should be adapted to the dose of epinephrine received during CPR. The development of strategies to prevent and control the severity of PRS in the population most at risk of death from cardiocirculatory causes should be considered for future randomized trials. For example, hypothermia at 33 °C was responsible for more arrhythmias resulting in hemodynamic compromise [35], while the optimal target for TTM in non-shockable rhythm OHCA is still under debate [7, 35, 36], as are blood pressure targets [37, 38]. In mild-to-moderate PRS, hypothermia at 33 °C does not seem to impact mortality [39, 40], but in the case of severe PRS leading to death, no data are available. Optimization of hemodynamics with a high blood pressure target and a TTM at 36 °C could be considered in subjects at risk of early death from cardiocirculatory causes after a non-shockable OHCA. In the era of personalized medicine, therapeutic targets will likely have to be adapted to each patient, particularly for management after ROSC. Indeed, one size does not fit all, and we suggest that it would be interesting for future randomized studies to take this epinephrine dose into account in order to personalize after ROSC management. In addition to being associated with the mode of death after ROSC, this variable is simple to collect and use routinely, unlike more complex scores, and its value seems highly reliable. Indeed, during CPR of a non-shockable rhythm, epinephrine is administered early in the procedure and at a regular frequency [9, 10]. Nevertheless, resuscitation times are imprecisely estimated during OHCA [30], and in addition, the timing (such as CPR time) reported in the databases is frequently inaccurate, aberrant, or missing [41–43]. The low-flow duration includes bystander CPR, BLS by rescuers, and ALS by MICU in different proportions depending on the timing and location of the OHCA. It is probably for this reason that we found only a weak correlation between low-flow duration and epinephrine doses during CPR and therefore adjusted our multivariable model on this variable and performed stratified analyses on the low-flow duration to asses a possible resuscitation time bias. We found the same effect of epinephrine on outcomes according to the duration of resuscitation, as well as a negative interaction factor in our model, reinforcing the idea that epinephrine dose during CPR was independently associated with outcomes.

Subsequent publications reported most post-cardiac arrest deaths were for neurological reasons (brain death and neurological WLST), whereas our study found a proportion of less than 50%. This is explained by the fact that we included only OHCA, unlike Witten et al. and Laver et al., who also included in-hospital cardiac arrest (IHCA) [18, 19]. Moreover, we only selected initial non-shockable rhythms, contrary to the other studies, which also included shockable rhythms, sometimes in the majority, as reported by Lemiale et al. [20]. Two-thirds of our cohort experienced post-resuscitation shock, and this accounted for a quarter of deaths, in agreement with the literature, which reports a prevalence of 50–70% with in-hospital mortality of 20–55% [15]. Neurological and cardiocirculatory causes were the most common modes of death in our study. However, for cumulative doses of epinephrine < 5 mg, neurological causes were more frequent, whereas after cumulative doses > 5 mg, cardiocirculatory causes were the predominant mode of death. Although epinephrine use was associated with all modes of death, this association was strongest between high cumulative doses and cardiocirculatory death.

### Limitations

This study had some limitations, notably due to the retrospective design, which does not establish a direct causal link between the dose of epinephrine administered during CPR and the mode of death after ROSC, but only an association. Second, in these studies, we could not analyze either the dose of epinephrine boluses or the timing of administration. Although practices are guided by the European Resuscitation Council (ERC) guidelines, i.e., administration of 1 mg of epinephrine every 3–5 min, we could not examine whether they were strictly followed in detail. Third, this study took place in a predominantly urban area, with relatively short response times for the MICU compared with more isolated rural areas (up to 30 min before ALS initiation), limiting the extrapolation of results. Fourth, subjects who received more than 5 mg of epinephrine had different characteristics (younger, more frequent OHCA of presumed cardiac cause) than those who received a lower dose; even though we adjusted our model on many covariates, it could not be excluded that other factors may have influenced the results.

## Conclusions

In our population admitted in ICU with ROSC, the dose of epinephrine used during CPR was strongly associated with early cardiocirculatory death in non-shockable OHCA. Strategies to prevent and manage post-resuscitation shock and sudden death recurrence might be evaluated in future randomized trials based on this cumulative dose of epinephrine. Furthermore, future studies limiting the dose of epinephrine during CPR also seem warranted.

### Supplementary Information

**Additional file 1.** **Table S1.** Characteristics of study population according to epinephrine dose administered during CPR CPR, cardiopulmonary resuscitation; PEA, pulseless electrical activity; CA, cardiac arrest; ECMO, extracorporeal membrane oxygenation; TTM, targeted temperature management **Table S2.** Multinomial regression after multiple data imputation (n = 2,792), reference population: alive at hospital discharge (full model) CPR, cardiopulmonary resuscitation; PEA, pulseless electrical activity; ECMO, extracorporeal membrane oxygenation; TTM, targeted temperature management. **Table S3.** Multinomial regression without multiple data imputation (n = 2,037), reference population: alive at hospital discharge Adjustment variables: gender, age, location, witnessed OHCA, bystander CPR, no-flow duration, initial electrical rhythm, low-flow duration, medical history: heart disease, cancer, renal disease, high blood pressure; ECMO use, coronary angioplasty intervention, TTM, post resuscitation shock, presumed cardiac cause. **Table S4.** Sensitivity analysis (+ years) after multiple data imputation (n = 2,792), reference population: alive at hospital discharge Adjustment variables: gender, age, location, witnessed OHCA, bystander CPR, no-flow duration, initial electrical rhythm, low-flow duration, medical history: heart disease, cancer, renal disease, high blood pressure; ECMO use, coronary angioplasty intervention, TTM, post resuscitation shock, presumed cardiac cause, years. **Table S5.** Sensitivity analysis (+ years) without multiple data imputation (n = 2,037), reference population: alive at hospital discharge. Adjustment variables: gender, age, location, witnessed OHCA, bystander CPR, no-flow duration, initial electrical rhythm, low-flow duration, medical history: heart disease, cancer, renal disease, high blood pressure; ECMO use,coronary angioplasty intervention, TTM, post resuscitation shock, presumed cardiac cause, years. **Table S6.** Sensitivity analysis, epinephrine as continuous variable, reference population: alive at hospital discharge Adjustment variables: gender, age, location, witnessed OHCA, bystander CPR, no-flow duration, initial electrical rhythm, low-flow duration, medical history: heart disease, cancer, renal disease, high blood pressure; ECMO use, coronary angioplasty intervention, TTM, post resuscitation shock, presumed cardiac cause, years. **Table S7.** Sensitivity analysis according to low-flow duration (tertiles), reference population: alive at hospital discharge Adjustment variables: gender, age, location, witnessed OHCA, bystander CPR, no-flow duration, initial electrical rhythm, low-flow duration, medical history: heart disease, cancer, renal disease, high blood pressure; ECMO use, coronary angioplasty intervention, TTM, post resuscitation shock, presumed cardiac cause. **Figure S1.** Linear regression of the low-flow duration (minutes) in relation to epinephrine dose used during CPR (mg). **Figure S2.** Linear regression of the proportion of deaths from cardiovascular causes (CCD, cardiovascular death) in relation to epinephrine dose used during CPR. **Figure S3.** Total epinephrine dose used during CPR in the complete population of the study. **Figure S4.** Percentage proportion of epinephrine use during CPR by outcome. **Figure S5.** Number of subjects by epinephrine dose used during CPR and outcome.

## Data Availability

The datasets used and/or analyzed during the current study are available from the corresponding author on reasonable request.

## Abbreviations

OHCA: Out-of-hospital cardiac arrest
IHCA: In-of-hospital cardiac arrest
ICU: Intensive care unit
CPR: Cardiopulmonary resuscitation
PRMD: Post-resuscitation myocardial dysfunction
ROSC: Return of spontaneous circulation
PRS: Post-resuscitation shock
WLST: Withdrawal of life-sustaining therapy
SDEC: Sudden Death Expertise Center
EMS: Emergency Medical Service
BLS: Basic life support
ALS: Advanced life support
MICU: Mobile intensive care unit
ECMO: Extracorporeal membrane oxygenation
TTM: Targeted temperature management
PEA: Pulseless electrical activity
aOR: Adjusted odds ratio
CI: Confidence interval
