# Supplementary material for: Cumulative dose of epinephrine and mode of death after non-shockable out-of-hospital cardiac arrest: a registry-based study
Source: Crit Care. 2023 Dec 20;27:496. doi: 10.1186/s13054-023-04776-0 (PMC10734153; doi:10.1186/s13054-023-04776-0)
Supplement: Supplementary file 1 — Additional file 1. Table S1. Characteristics of study population according to epinephrine dose administered during CPR CPR, cardiopulmonary resuscitation; PEA, pulseless electrical activity; CA, cardiac arrest; ECMO, extracorporeal membrane oxygenation; TTM, targeted temperature management Table S2. Multinomial regression after multiple data imputation (n = 2,792), reference population: alive at hospital discharge (full model) CPR, cardiopulmonary resuscitation; PEA, pulseless electrical activity; ECMO, extracorporeal membrane oxygenation; TTM, targeted temperature management. Table S3. Multinomial regression without multiple data imputation (n = 2,037), reference population: alive at hospital discharge Adjustment variables: gender, age, location, witnessed OHCA, bystander CPR, no-flow duration, initial electrical rhythm, low-flow duration, medical history: heart disease, cancer, renal disease, high blood pressure; ECMO use, coronary angioplasty intervention, TTM, post resuscitation shock, presumed cardiac cause. Table S4. Sensitivity analysis (+ years) after multiple data imputation (n = 2,792), reference population: alive at hospital discharge Adjustment variables: gender, age, location, witnessed OHCA, bystander CPR, no-flow duration, initial electrical rhythm, low-flow duration, medical history: heart disease, cancer, renal disease, high blood pressure; ECMO use, coronary angioplasty intervention, TTM, post resuscitation shock, presumed cardiac cause, years. Table S5. Sensitivity analysis (+ years) without multiple data imputation (n = 2,037), reference population: alive at hospital discharge. Adjustment variables: gender, age, location, witnessed OHCA, bystander CPR, no-flow duration, initial electrical rhythm, low-flow duration, medical history: heart disease, cancer, renal disease, high blood pressure; ECMO use,coronary angioplasty intervention, TTM, post resuscitation shock, presumed cardiac cause, years. Table S6. Sensitivity analysis, epinephrine as contin [file 13054_2023_4776_MOESM1_ESM.pdf]

Additional file 1

Table S1

Characteristics of study population according to epinephrine dose administered during CPR

**Variables**<br>**n (%) or mean ± SD**	**0 mg**<br>**(n = 243)**	**1 mg**<br>**(n = 445)**	**2 – 5 mg**<br>**(n = 1586)**	**> 5 mg**<br>**(n = 518)**	***p-value***<br>**Chi2 or ANOVA**
Male	138 (56.8%)	272 (61.1%)	958 (60.4%)	334 (64.5%)	0.20
Age (years)	67.3 ± 17.3	64.3 ± 16.1	63.8 ± 14.8	59.8 ± 14.6	< 0.001
Home location	181 (74.5%)	316 (71.2%)	1159 (73.1%)	353 (68.3%)	0.15
Witnessed	210 (86.4%)	366 (82.4%)	1297 (82.0%)	440 (84.9%)	0.20
Bystander CPR	141 (58.8%)	233 (52.6%)	902 (57.6%)	335 (64.9%)	0.001
No-flow duration (min)	4.1 ± 5.0	7.1 ± 9.3	6.9 ± 8.4	5.2 ± 7.1	< 0.001
First recorded rhythm					< 0.001
Asystole	193 (79.4%)	352 (79.1%)	1369 (86.3%)	447 (86.3%)	
PEA	50 (20.6%)	93 (20.9%)	217 (13.7%)	71 (13.7%)	
Low-flow duration (min)	15.5 ± 15.9	21.6 ± 13.5	27.6 ± 16.5	49.3 ± 30.9	< 0.001
Heart disease	76 (31.3%)	130 (29.2%)	464 (29.3%)	137 (26.5%)	0.51
Ischemic cardiopathy	39 (16.1%)	69 (15.5%)	243 (15.3%)	65 (12.6%)	0.41
Cancer	37 (15.2%)	55 (12.4%)	192 (12.1%)	34 (6.6%)	0.001
Renal disease	19 (7.8%)	38 (8.5%)	151 (9.5%)	33 (6.4%)	0.16
High blood pressure	103 (42.4%)	174 (39.1%)	689 (43.4%)	213 (41.1%)	0.39
Diabetes mellitus	32 (13.2%)	81 (18.2%)	392 (24.7%)	143 (27.6%)	< 0.001
Dyslipidemia	35 (14.4%)	72 (16.2%)	272 (17.2%)	94 (18.2%)	0.60
ECMO	11 (4.5%)	10 (2.3%)	97 (6.1%)	112 (21.6%)	< 0.001
Shock	4 (36.4%)	4 (40.0%)	21 (21.7%)	7 (6.2%)	
ARDS	0 (0.0%)	0 (0.0%)	1 (1.0%)	0 (0.0%)	
Refractory CA	7 (63.6%)	6 (60.0%)	75 (77.3%)	105 (93.8%)	
Coronary angioplasty	20 (8.2%)	40 (9.0%)	142 (9.0%)	54 (10.4%)	0.72
TTM	77 (32.6%)	202 (46.1%)	645 (41.5%)	171 (33.7%)	< 0.001
Post-resuscitation shock	118 (51.8%)	282 (66.7%)	1072 (70.9%)	370 (79.1%)	< 0.001
Presumed cause of CA					< 0.001
Cardiac	108 (44.4%)	209 (47.0%)	773 (48.7%)	321 (62.0%)	
Non-cardiac	135 (55.6%)	236 (53.0%)	813 (51.3%)	197 (38.0%)	
Outcomes					< 0.001
Alive	90 (37.1%)	57 (12.8%)	83 (5.2%)	12 (2.3%)	
Cardiocirculatory death	43 (17.7%)	100 (22.5%)	575 (36.3%)	286 (55.2%)	
Neurological death	72 (29.6%)	236 (53.0%)	750 (47.3%)	175 (33.8%)	
Other death	38 (15.6%)	52 (11.7%)	178 (11.2%)	45 (8.7%)	

CPR, cardiopulmonary resuscitation; PEA, pulseless electrical activity; CA, cardiac arrest; ECMO, extracorporeal membrane oxygenation; TTM, targeted temperature management

Table S2. Multinomial regression after multiple data imputation (n = 2,792), reference population: alive at hospital discharge (full model)

Reference outcome = alive	Cardiocirculatory death		Neurological death		Other death	
	aOR CI95%	p-value	aOR CI95%	p-value	aOR CI95%	p-value
Sex male	0.82 (0.58-1.16)	0.26	0.73 (0.53-1.02)	0.064	0.75 (0.51-1.11)	0.15
Age (per year)	1.04 (1.03-1.05)	<0.001	1.01 (1.00-1.03)	0.015	1.06 (1.04-1.07)	<0.001
Home location	1.57 (1.11-2.24)	0.011	1.25 (0.90-1.73)	0.19	1.96 (1.28-3.00)	0.002
Witnessed	1.69 (0.95-3.03)	0.076	1.51 (0.87-2.62)	0.15	2.24 (1.14-4.38)	0.019
Bystander CPR	0.97 (0.60-1.56)	0.90	0.77 (0.48-1.21)	0.25	0.99 (0.58-1.68)	0.97
No flow duration (per min)	1.08 (1.04-1.12)	<0.001	1.08 (1.04-1.11)	<0.001	1.08 (1.04-1.12)	<0.001
Asystole	Reference		Reference		Reference	
PEA	1.09 (0.72-1.64)	0.68	0.81 (0.55-1.21)	0.30	0.70 (0.43-1.16)	0.17
Epinephrine during CPR						
0 mg	Reference		Reference		Reference	
1 mg	3.45 (2.01-5.92)	<0.001	4.40 (2.79-6.94)	<0.001	2.69 (1.49-4.85)	0.001
2-5 mg	12.28 (7.52-20.06)	<0.001	8.88 (5.80-13.59)	<0.001	5.95 (3.49-10.13)	<0.001
>5 mg	23.71 (11.02-50.97)	<0.001	10.26 (4.94-21.31)	<0.001	7.45 (3.20-17.33)	<0.001
Low flow duration (per min)	1.04 (1.03-1.06)	<0.001	1.04 (1.02-1.05)	<0.001	1.04 (1.02-1.05)	<0.001
Heart disease	0.86 (0.58-1.28)	0.46	1.04 (0.71-1.51)	0.84	1.08 (0.70-1.67)	0.73
Cancer	0.99 (0.59-1.67)	0.98	1.10 (0.67-1.81)	0.69	1.74 (1.01-3.01)	0.047
Renal disease	1.27 (0.68-2.36)	0.45	1.23 (0.69-2.21)	0.49	1.44 (0.74-2.80)	0.29
High blood pressure	0.55 (0.38-0.79)	0.001	0.76 (0.54-1.08)	0.13	0.58 (0.38-0.87)	0.008
ECMO	0.57 (0.27-1.18)	0.13	0.35 (0.17-0.73)	0.005	0.88 (0.37-2.12)	0.78
Coronary angioplasty	0.51 (0.30-0.86)	0.012	0.51 (0.31-0.85)	0.010	0.42 (0.22-0.79)	0.008
TTM	0.25 (0.18-0.35)	<0.001	0.83 (0.61-1.14)	0.25	0.30 (0.20-0.45)	<0.001
Post resuscitation shock	2.13 (1.48-3.05)	<0.001	0.78 (0.56-1.08)	0.13	0.86 (0.58-1.28)	0.46
Presumed cardiac cause	2.44 (1.69-3.52)	<0.001	1.06 (0.74-1.50)	0.75	1.80 (1.20-2.71)	0.005

CPR, cardiopulmonary resuscitation; PEA, pulseless electrical activity; ECMO, extracorporeal membrane oxygenation; TTM, targeted temperature management

Table S3. Multinomial regression without multiple data imputation (n = 2,037), reference population: alive at hospital discharge

Reference outcome = alive	Cardiocirculatory death		Neurological death		Other death	
	**aOR CI95%**	**p-value**	**aOR CI95%**	**p-value**	**aOR CI95%**	**p-value**
Epinephrine during CPR						
0 mg	Reference		Reference		Reference	
1 mg	3.30 (1.74-6.26)	<0.001	3.75 (2.20-6.39)	<0.001	2.64 (1.33-5.23)	0.005
2-5 mg	11.99 (6.62-21.68)	<0.001	8.41 (5.08-13.91)	<0.001	5.87 (3.12-11.03)	<0.001
>5 mg	19.06 (7.92-45.87)	<0.001	9.16 (4.02-20.86)	<0.001	5.61 (2.11-14.89)	0.001

Adjustment variables: gender, age, location, witnessed OHCA, bystander CPR, no-flow duration, initial electrical rhythm, low-flow duration, medical history: heart disease, cancer, renal disease, high blood pressure; ECMO use, coronary angioplasty intervention, TTM, post resuscitation shock, presumed cardiac cause.

Table S4. Sensitivity analysis (+ years) after multiple data imputation (n = 2,792), reference population: alive at hospital discharge

**Reference outcome = alive**	**Cardiocirculatory death**		**Neurological death**		**Other death**	
	**aOR CI95%**	**p-value**	**aOR CI95%**	**p-value**	**aOR CI95%**	**p-value**
Epinephrine during CPR						
0 mg	Reference		Reference		Reference	
1 mg	3.49 (2.03-5.99)	<0.001	4.50 (2.85-7.10)	<0.001	2.73 (1.51-4.94)	0.001
2-5 mg	12.39 (7.58-20.26)	<0.001	9.02 (5.89-13.82)	<0.001	6.02 (3.53-10.25)	<0.001
>5 mg	23.76 (11.05-51.11)	<0.001	10.19 (4.91-21.17)	<0.001	7.41 (3.19-17.25)	<0.001
Years (per year)	0.99 (0.94-1.04)	0.65	0.97 (0.92-1.02)	0.18	0.97 (0.91-1.04)	0.41

Adjustment variables: gender, age, location, witnessed OHCA, bystander CPR, no-flow duration, initial electrical rhythm, low-flow duration, medical history: heart disease, cancer, renal disease, high blood pressure; ECMO use, coronary angioplasty intervention, TTM, post resuscitation shock, presumed cardiac cause, years.

Table S5. Sensitivity analysis (+ years) without multiple data imputation (n = 2,037), reference population: alive at hospital discharge

Reference outcome = alive	Cardiocirculatory death		Neurological death		Other death	
	**aOR CI95%**	**p-value**	**aOR CI95%**	**p-value**	**aOR CI95%**	**p-value**
Epinephrine during CPR						
0 mg	Reference		Reference		Reference	
1 mg	3.34 (1.76-6.35)	<0.001	3.81 (2.24-6.50)	<0.001	2.68 (1.35-5.31)	0.005
2-5 mg	12.08 (6.67-21.86)	<0.001	8.48 (5.12-14.04)	<0.001	5.90 (3.14-11.10)	<0.001
>5 mg	19.11 (7.93-46.06)	<0.001	9.06 (3.98-20.65)	<0.001	5.55 (2.09-14.76)	0.001
Years (per year)	0.99 (0.93-1.06)	0.75	0.97 (0.91-1.03)	0.34	0.98 (0.91-1.05)	0.51

Adjustment variables: gender, age, location, witnessed OHCA, bystander CPR, no-flow duration, initial electrical rhythm, low-flow duration, medical history: heart disease, cancer, renal disease, high blood pressure; ECMO use, coronary angioplasty intervention, TTM, post resuscitation shock, presumed cardiac cause, years.

Table S6. Sensitivity analysis, epinephrine as continuous variable, reference population: alive at hospital discharge

Reference outcome = alive	Cardiocirculatory death		Neurological death		Other death	
	**aOR CI95%**	**p-value**	**aOR CI95%**	**p-value**	**aOR CI95%**	**p-value**
Epinephrine during CPR, per mg	1.46 (1.33-1.61)	<0.001	1.33 (1.21-1.46)	<0.001	1.41 (1.27-1.57)	0.001

Adjustment variables: gender, age, location, witnessed OHCA, bystander CPR, no-flow duration, initial electrical rhythm, low-flow duration, medical history: heart disease, cancer, renal disease, high blood pressure; ECMO use, coronary angioplasty intervention, TTM, post resuscitation shock, presumed cardiac cause, years.

Table S7. Sensitivity analysis according to low-flow duration (tertiles), reference population: alive at hospital discharge

	**Cardiocirculatory death**		**Neurological death**		**Other death**	
	**aOR 95%CI**	**p-value**	**aOR 95%CI**	**p-value**	**aOR 95%CI**	**p-value**
**Low-flow duration ≤ 19 min (n = 868)**						
Epinephrine during CPR						
0 mg	Reference		Reference		Reference	
1 mg	2.53 (1.27-5.06)	0.009	4.08 (2.34-7.12)	<0.001	2.14 (0.99-4.63)	0.053
2-5 mg	12.96 (6.96-24.15)	<0.001	8.26 (4.82-14.15)	<0.001	5.13 (2.56-10.26)	<0.001
> 5 mg	12.06 (3.76-38.69)	<0.001	5.42 (1.73-16.93)	0.004	5.64 (1.41-22.64)	0.015
**Low-flow duration 20 min to 30 min (n = 1,023)**						
Epinephrine during CPR						
0 mg	Reference		Reference		Reference	
1 mg	4.13 (1.46-11.71)	0.008	4.48 (1.81-11.08)	0.001	4.23 (1.36-13.11)	0.013
2-5 mg	12.06 (4.67-31.13)	<0.001	8.58 (3.78-19.47)	<0.001	6.53 (2.34-18.23)	<0.001
> 5 mg	44.58 (8.33-238.62)	<0.001	22.36 (4.48-111.50)	<0.001	14.89 (2.44-90.85)	0.003
**Low-flow duration > 30 min (n = 901)**						
Epinephrine during CPR						
0 mg	Reference		Reference		Reference	
1 mg	4.63 (0.40-53.30)	0.219	4.99 (0.44-56.65)	0.195	3.83 (0.18-81.17)	0.388
2-5 mg	12.86 (1.52-108.85)	0.019	7.76 (0.91-66.41)	0.062	9.74 (0.63-149.99)	0.103
> 5 mg	52.76 (5.81-479.46)	<0.001	17.67 (1.92-162.62)	0.011	21.87 (1.33-358.93)	0.031

Adjustment variables: gender, age, location, witnessed OHCA, bystander CPR, no-flow duration, initial electrical rhythm, low-flow duration, medical history: heart disease, cancer, renal disease, high blood pressure; ECMO use, coronary angioplasty intervention, TTM, post resuscitation shock, presumed cardiac cause.

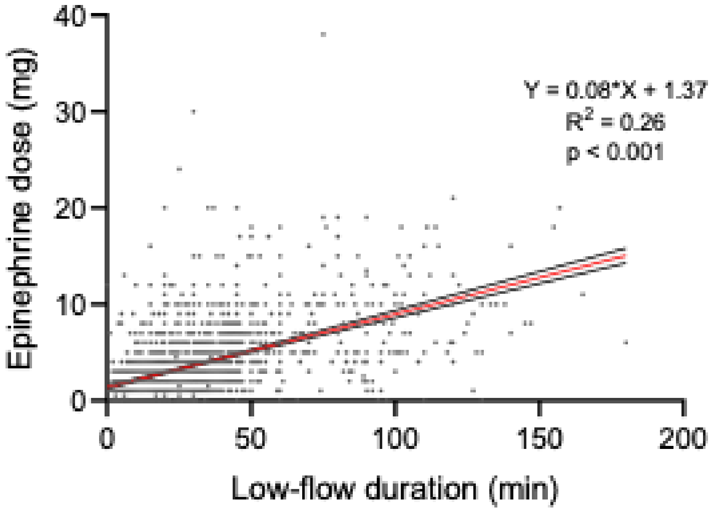

Figure S1. Linear regression of the low-flow duration (minutes) in relation to epinephrine dose used during CPR (mg).

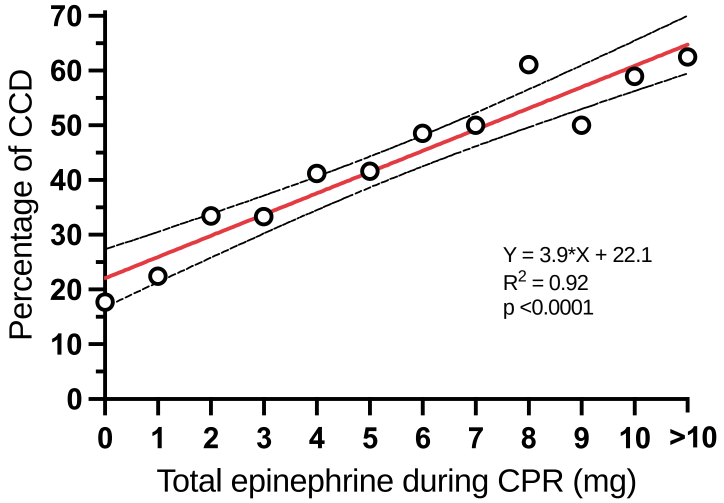

Figure S2. Linear regression of the proportion of deaths from cardiovascular causes (CCD, cardiovascular death) in relation to epinephrine dose used during CPR.

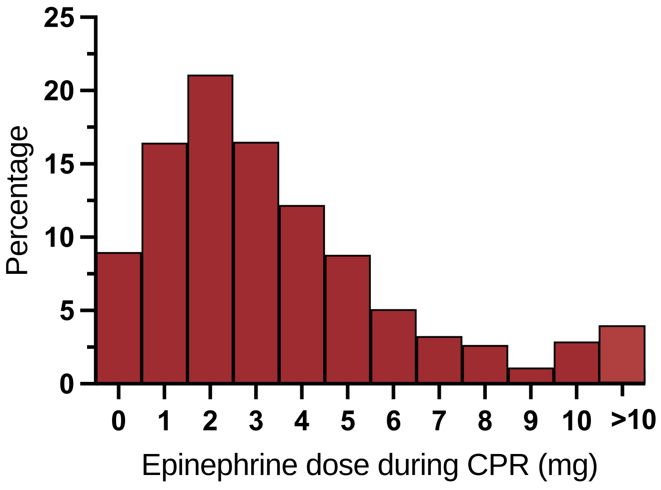

Figure S3. Total epinephrine dose used during CPR in the complete population of the study.

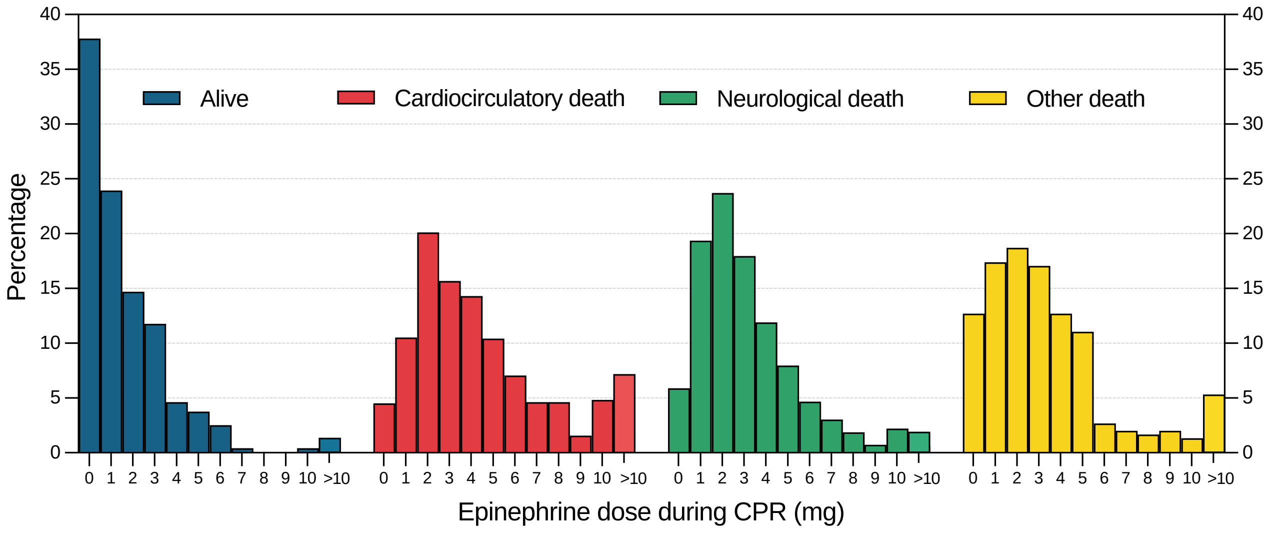

Figure S4. Percentage proportion of epinephrine use during CPR by outcome.

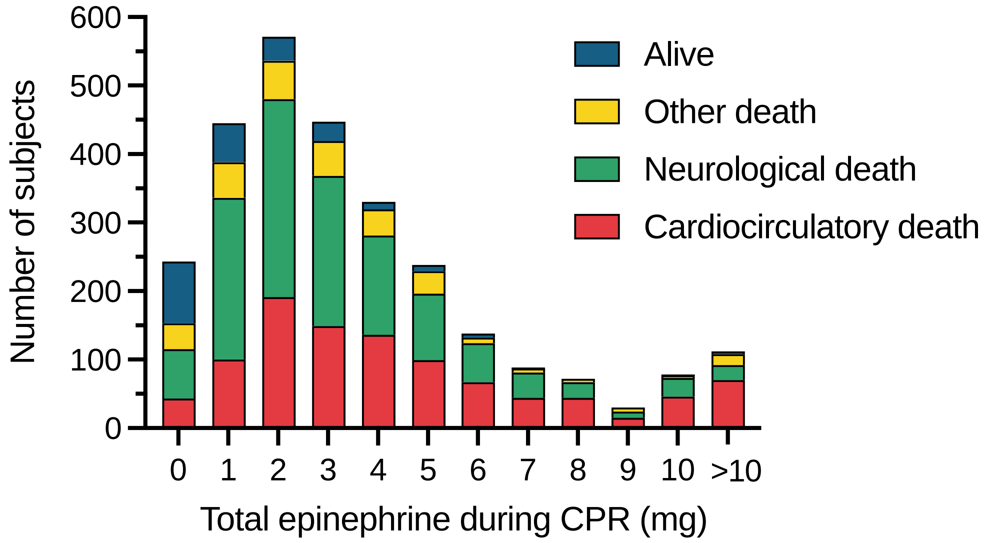

Figure S5. Number of subjects by epinephrine dose used during CPR and outcome.
